# Brain Networks during Free Viewing of Complex Erotic Movie: New Insights on Psychogenic Erectile Dysfunction

**DOI:** 10.1371/journal.pone.0105336

**Published:** 2014-08-15

**Authors:** Nicoletta Cera, Ezio Domenico Di Pierro, Antonio Ferretti, Armando Tartaro, Gian Luca Romani, Mauro Gianni Perrucci

**Affiliations:** 1 Department of Neuroscience, Imaging and Clinical Science, “G.d’Annunzio” University of Chieti, and ITAB–Institute for Advanced Biomedical Technologies, Chieti, Italy; 2 Division of Urology, “L’immacolata” Hospital of Celano, Celano, Italy; 3 Catholic University of the Sacred Heart, Rome, Italy; West China Hospital of Sichuan University, China

## Abstract

Psychogenic erectile dysfunction (ED) is defined as a male sexual dysfunction characterized by a persistent or recurrent inability to attain adequate penile erection due predominantly or exclusively to psychological or interpersonal factors. Previous fMRI studies were based on the common occurrence in the male sexual behaviour represented by the sexual arousal and penile erection related to viewing of erotic movies. However, there is no experimental evidence of altered brain networks in psychogenic ED patients (EDp). Some studies showed that fMRI activity collected during non sexual movie viewing can be analyzed in a reliable manner with independent component analysis (ICA) and that the resulting brain networks are consistent with previous resting state neuroimaging studies. In the present study, we investigated the modification of the brain networks in EDp compared to healthy controls (HC), using whole-brain fMRI during free viewing of an erotic video clip. Sixteen EDp and nineteen HC were recruited after RigiScan evaluation, psychiatric, and general medical evaluations. The performed ICA showed that visual network (VN), default-mode network (DMN), fronto-parietal network (FPN) and salience network (SN) were spatially consistent across EDp and HC. However, between-group differences in functional connectivity were observed in the DMN and in the SN. In the DMN, EDp showed decreased connectivity values in the inferior parietal lobes, posterior cingulate cortex and medial prefrontal cortex, whereas in the SN decreased and increased connectivity was observed in the right insula and in the anterior cingulate cortex respectively. The decreased levels of intrinsic functional connectivity principally involved the subsystem of DMN relevant for the self relevant mental simulation that concerns remembering of past experiences, thinking to the future and conceiving the viewpoint of the other’s actions. Moreover, the between group differences in the SN nodes suggested a decreased recognition of autonomical and sexual arousal changes in EDp.

## Introduction

Current guidelines have defined Psychogenic Erectile Dysfunction (ED) as the male sexual dysfunction characterized by a persistent or recurrent inability to attain, or maintain adequate penile erection until completion of sexual activity, caused predominantly or exclusively by psychological or interpersonal factors [1], [2]. Several psychological factors are related to the development of ED. Particularly, traumatic past experiences, inadequate sex education and strict upbringing can be considered predisposing factors. However, during the lifespan, relationship problems, family or social pressures and major life events can be considered precipitating factors for psychogenic ED [3]. Moreover, Psychogenic ED induces marked distress or interpersonal difficulty (DSM-IV). In daily clinical practice, psychogenic ED patients report a series of couple relationship problems, interpersonal difficulty and stress associated to important life events, such as lost of job or economical problems.

In the last ten years, several neuroimaging studies, conducted in healthy volunteers using visual sexual stimulation described a complex set of cortical and subcortical brain regions, such as the Anterior and Middle Cingulate Cortex (ACC; MCC), the Insula, the Claustrum, and the Hypothalamus [4]–[18]. Conversely, few studies investigated brain activity in patients with psychogenic ED (EDp) compared to healthy controls (HC) [8], [11]. Furthermore, only one study showed a decreased volume of gray matter in correspondence of the Ventral Striatum and Hypothalamus in EDp compared to HC [19].

In the last decade, fMRI studies showed regional differences in the BOLD activity comparing the presentation of sexual to non-sexual visual stimuli [4]–[18]. The basis of these imaging studies is the concurrent recording of penile erection, considered a marker of the sexual arousal and a common occurrence in the male sexual behaviour during visual erotic stimulation [4], [5].

Recently, there has been a growing interest for the brain activity elicited by ecologically-valid stimuli [18], [20]–[22]. The central issue in the activation paradigms is the straightforward correlation between the stimuli presented to the subject and some specific brain function. Hypothesis-based analysis methods, such as General Linear Model (GLM), cannot be applied to the data collected during complex cinematographic stimulation [23]. Indeed, previous studies, using dynamical non-sexual cinematographic material, have applied data-driven approaches that do not require any “a priori” hypothesis. These studies have demonstrated that complex fMRI data can be reliably analyzed by means of the Independent Component Analysis (ICA) showing consistent results [21], [22]. Thus, ICA is a useful tool to analyze fMRI data collected during cinematographic stimulation [21], [22]. Once collected, ICA can separate the fMRI data into additive and spatially independent components. This approach is based on the intrinsic structure of the data, without any “a priori” assumption. The resulting brain networks summarize the functional architecture of somato-motor, visual, auditory, attention, language, and memory networks that are commonly modulated during active behavioral tasks [24], [25]. Most of the knowledge about processes underlying the brain networks came from resting state fMRI (rsfMRI) studies.

The most studied brain networks are: the Default Mode Network (DMN) [26], the Salience Network (SN); the Fronto Parietal Control Network (FPN), the primary Sensory Motor Network (SMN), the Visual Network (VN), and the Dorsal Attention Network (DAN) [27]–[28]. These networks are related to the main sensory, cognitive and emotional functions [26]–[31].

The male sexual arousal can be conceived as a multidimensional experience comprising sensory, autonomic, cognitive, and emotive components [5], [7]. On the other hand, sexual inhibition, considered a very complex set of processes, is one of the most important features of Psychogenic ED.

From our previous study [11], Psychogenic ED seems to be related to the aberrant appraisal of the erotic stimuli, the self–awareness of the body changes, compromising the high level processing. This aberrant brain response has been related to the activity observed in regions as the medial Prefrontal Cortex (mPFC), the Parietal Lobes, the Insular, and the ACC/MCC that are critical nodes for the DMN, the FPN, and the SN [26]–[31].

In the present fMRI study, EDp and HC were presented with an erotic video clip to assess the topological differences in brain networks, using ICA. Free viewing of the selected erotic clip brings sexual arousal in healthy men, allowing the investigation of brain networks related to the normal and the pathological sexual behaviour.

## Materials and Methods

### Subjects and stimulus

Sixteen right handed heterosexual outpatients affected by Psychogenic Erectile Dysfunction (mean age 33.4±10.7 SD, range 19–63) (EDp) and nineteen healthy right handed heterosexual men (mean age = 33.5±11.4 SD, range 21–67) (HC) were included in the study.

The diagnosis of Psychogenic Erectile dysfunction was performed according to the following criteria: absence of organic comorbidities or vascular risk factors for Erectile Dysfunction, normal morning erections, normal penile hemodynamics according to the color Doppler Sonography and normal nocturnal erections as evaluated by the RigiScan® device during three consecutive nights. Normal nocturnal erections and penile hemodynamic were verified also in HC, revealing similar values for the two groups.

Exclusion criteria for both groups were: (i) meeting DSM-IV criteria for any 1 and 2 axis disorders assessed with interviewer-administered Mini-International Neuropsychiatric Interview (M.I.N.I.) [32]; (ii) use of any psychoactive medication and other medications that might affect sexual function; (iii) use of recreational drugs during the previous 30 days; (iv) use of medication designed to enhance sexual performance; and (v) history of committing any sexual offence assessed by means of a clinical anamnestic interview.

For HC, additional exclusion criteria were as follow: (i) history of erectile dysfunction; (ii) lacking experience of sexual intercourse.

EDp and HC (Table 1) did not differ in ethnicity, age, education, marital and socioeconomic status, and nicotine use [33].

**Table 1:** Psychological and behavioral results.

Demographics					
		EDp(n = 16)	HC(n = 19)	EDp vs HC	
		M ± SD	M ± SD	t value	*P*
**Age (Yrs)**		33.87±11.2	33.57±11.4	0.076	0.939
**Caucasian**		16	19		
**Education (Yrs)**		14.5±2.3	14.5±2.7	0.041	0.967
**Duration (mths)**		9.2±5.1	-	-	-
**Smokers**		7	11		
**Number of cigarettes per day**		4.85±1.71	5.57±1.38	0.322	0.608
**Married/cohabitation**		14	17		
**Psychological data**					
		**M ± SD**	**M ± SD**	**t value**	***P***
**IIEF −5**		14.5±5.5	24±0.93	−7.37	<0.0001
**BIS BAS Scale**					
	BIS	20.8±3.4	21.26±3.7	−0.34	0.729
	BAS RewardResponsiveness	19±2.4	19.6±2.5	−0.77	0.444
	BAS Drive	12±3.7	12.8±2.7	−0.75	0.454
	BAS Fun Seeking	11.2±2.8	13±2.3	−2.03	<0.05
**STAI**					
	STAI Y1	38.1±8.9	35.4±7.3	0.98	0.332
	STAI Y2	43.2±9.2	39±8.5	1.41	0.165
**SAI-E**					
	SAI (Excitation)	74±26.7	106±28.1	−4.03	<0.001
	SAI (Anxiety)	76±17.4	98±14.8	1.90	0.0651
	SAI tot scores	2.06±15.5	−7.84±15.3	−3.42	<0.005
**Physiological data**					
		**M ± SD**	**M ± SD**	**t value**	***P***
**Penile tumescence** *(percent var.)*		0.81±7.1	10.32±16.4	−2.13	<0.05
**Heart rate** *(beats/min)*		74±12.5	75.4±11.2	0.35	0.725
**Respiratory rate** *(cycles/min)*		0.68±0.10	0.69±0.17	0.16	0.871

Data from demographics, questionnaires and physiological measures are presented in terms of mean score (M) and standard deviation (SD) in EDp and HC group. Statistical comparisons between the two groups are provided.

EDp: Erectile Dysfunction patients; HC: healthy Controls; IIEF-5: International Index of Erectile Function; STAI: State Trait Anxiety Inventory Y form; BIS BAS scale: Behavioural Inhibition System and Behavioural Activation System Scale; SAI-E: Sexual Arousability Inventory Expanded. The *t* and *p* values were obtained by two tailed *t* test.

All potential subjects underwent a 1-h interview with a psychiatrist and filled out a number of questionnaires including the International Index of Erectile Function (IIEF) [34], Sexual Arousal Inventory Expanded (SAI-E) [35], [36] and SCL-90-R [37], State-Trait Anxiety Inventory (STAI) [38], BIS/BAS scale [39]. The study design was explained in detail and all subjects read and signed an informed consent form prior to being interviewed and filling out the questionnaires. Subjects’ consent was obtained according to the Helsinki Declaration. The study was approved by the University of Chieti ethics committee.

For the selection of video stimuli, 30 erotic clips were chosen from commercial movies and presented in a randomized order to 20 healthy subjects (age 20–61 years) that privately viewed and rated the excerpts, according to a rating scale, with a minimum of 1 and a maximum of 7, considering the following dimensions: quality of clips and perceived arousal. However the subjects recruited for stimulus selection did not take part in the fMRI experiment.

Each selected video clip showed consensual sexual interactions between one man and one woman (petting, vaginal intercourse, and oral sex) following the guidelines of Koukounas and Over [40] and was presented to each participant for 7 minutes.

Subjects were asked to report their feeling of sexual arousal by pressing an MRI compatible button at the beginning of his perceived sexual arousal.

The video clip presentation and the button press recording were controlled by a MATLAB home made program running on a PC placed in the scanner console room. Erotic movie was projected on a translucent glass placed on the back of the scanner bore by means of an LCD projector. A mirror fixed to the head coil inside the magnet allowed the subjects to view the clip.

At the end of fMRI session, each subject was asked about his feeling of sexual arousal during the viewing of the clip according to a 7-points rating scale (1 extremely low to 7 extremely high).

### Physiological monitoring

Penile tumescence was continuously recorded during movie presentation and fMRI data acquisition by means of a custom-built MRI-compatible pneumatic device based on a newborn sized blood pressure cuff. Prior to the start of the fMRI acquisition, the pressure cuff was placed on the penis using a condom and was inflated to an initial pressure of 80 mm Hg. The cuff was connected by a thin tube to a pressure transducer placed in the console room. The pressure transducer was connected to an amplifier and the analog signal from this device was recorded at a sampling rate of 100 Hz on a PC for off-line data analysis.

A scanner’s built in photoplethysmograph placed on the left index finger monitored the heart signals, while a pneumatic respiratory belt was strapped around the upper abdomen to measure the expansion of the subject’s respiration. Both cardio and respiratory (CR) signals were sampled by the scanner at 100 Hz and stored in a file in txt format. Moreover the heart beat signal was marked each time an R-peak was detected.

### fMRI data acquisition

Functional and structural imaging was performed with a 3T Philips Achieva MRI scanner (Philips Medical Systems, Best, The Netherlands) using a whole-body radiofrequency coil for signal excitation and an eight-channel head coil for signal reception. Blood Oxygen Level Dependent (BOLD) fMRI data were acquired by means of T2*-weighted echo-planar (EPI) sequences with the following parameters: TE = 35 ms, matrix size = 80×80, FOV = 230 mm, in-plane voxel size = 2.875×2.875 mm, SENSE factor 1.8 anterior-posterior, flip angle = 80°, slice thickness = 3 mm with no gap. During the session 210 functional volumes consisting of 31 transaxial slices were acquired with a TR of 2 s.

A high resolution structural volume was acquired at the end of the session via a 3D fast field echo T1-weighted sequence (voxel size 1 mm isotropic, TR/TE = 8.1/3.7 ms; flip angle 8°, SENSE factor 2).

### Data analysis

Penile tumescence time series were down-sampled from 100 Hz to the sampling rate of the functional MRI volumes (TR = 2 s), linear detrended and transformed in percent change values. Average percent change normalized penile tumescence (PT) value was calculated for the whole period of visual stimulation for each subject and compared between groups by means of a two tailed t-test.

Cardiac and respiratory rate time series were calculated and resampled to the TR value using a home made program implemented in MATLAB (The MathWorks Inc., Natick, MA, USA). Average cardiac rate (HR) and respiratory rate (RR) values for visual stimulation were obtained for each subject. Statistically significant between groups differences in HR and RR were assessed by means of a two-tailed t-test (Group: EDp, HC).

BOLD fMRI data were analyzed by means of the Brain Voyager QX software (Brain Innovation, The Netherlands).

Due to T1 saturation effects, the first 2 scans of each run were discarded from the analysis. Preprocessing of functional scans included motion correction, removal of linear trends from voxel time series and slice scan-time correction. To match each functional volume to the reference volume, the motion correction was performed by three-dimensional rigid body transformation. The estimated translation and rotation parameters for each volume in the time course were inspected to check that the movement was not larger than approximately half a voxel [41], [42]. Then, they were spatially smoothed by convolution with an isotropic Gaussian kernel (FWHM = 6 mm).

Preprocessed functional volumes of a subject were coregistered with the corresponding structural data set. Since the 2D functional and 3D structural measurements were acquired in the same session, the coregistration transformation was determined using the position parameters of the structural volume. The alignment between functional and anatomical scans was finally checked by means of an accurate visual inspection. Structural and functional volumes were transformed into Talairach space [43] using a piecewise affine and continuous transformation. Functional volumes were resampled at a voxel size of 3×3×3 mm^3^.

We included 2 covariates that modelled signals sampled from White Matter (WM) and Cerebro-Spinal Fluid (CSF) [44]. We derived the WM and CSF signals averaging the time courses of the voxels in each subject’s WM masks and CSF. The WM masks were generated by the segmentation process of each subject’s brain, while CSF signals were sampled from the third ventricle of each subject’ brain.

Spatial ICA was used to analyze functional MR imaging data sets for the decomposition of the voxel time series into a set of independent spatiotemporal patterns (ICs).

Using the FastICA algorithm, we estimated 30 ICs for each subject [45], with a deflation approach and tanh nonlinearity [46], [47], consistently also with guidelines proposed by Pamilo and colleagues [21]. To select ICs of interest, we used templates of intrinsic connectivity networks (ICNs) within the brain from previous published papers. Spatial cross correlation was performed for each template. The brain networks templates from previous studies [46], [47] considered in the current work were: Fronto Parietal Network (FPN), Central Executive Network (CEN), Default Mode Network (DMN), Somato-Motor Network (SMN), Visual Network (VN), Auditory Network (AN), and Salience Network (SN).

To extend the ICA analysis from single-subject to multisubject studies, the ICs estimated from each subject were clustered with self-organizing group ICA (sogICA) method, according to their mutual similarities [24]. A reduced number of spatio-temporally distinct patterns of low-frequency fluctuations were extracted [46], [47].

As ICA on fMRI data intrinsically extracts patterns of coherent neuronal activity (i.e. networks), Z values, obtained from individual maps, can indirectly provide a measure of functional connectivity within the network [48].

For each network, between-groups differences were assessed by means of a voxel-wise one way ANOVA on Z values, obtained by individual ICA group maps, and as clusters of interest were considered only those included in the nodes of each ICNs.

Between group difference maps were thresholded at a significance level (the probability of a false detection for the entire functional volume) of α<0.05, corrected for multiple comparisons. The correction for multiple comparisons was performed using a cluster-size thresholding algorithm [49] based on Monte Carlo simulations implemented in the BrainVoyager QX software. A threshold of p<0.005 at the voxel level, a FWHM = 1.842 voxel as Gaussian kernel of the spatial correlation among voxels and 5000 iterations were used as input in the simulations, yielding a minimum cluster size of 22 voxels.

After voxel wise analysis, the Z values are extrapolated from the clusters of the maps, showing a between group difference, and a two tailed t test was performed.

Moreover, a Pearson’s correlation analysis was performed to investigate the relationship between Z values from the network maps and the sexual functions as measured by SAI-E and IIEF.

Specifically, the mean Z values of each ROI were correlated to IIEF and SAI-E (including total score, Excitation and Anxiety subscale scores).

## Results

### Behavioural and physiological data

Sociodemographic, psychological and behavioural data of EDp and HC are presented in Table1.

Between-group differences on education (years) and age were not significant. In EDp, scores on Sexual Arousal Inventory and on the IIEF were significantly lower than those of healthy volunteers (Table 1).

**Figure 1:**
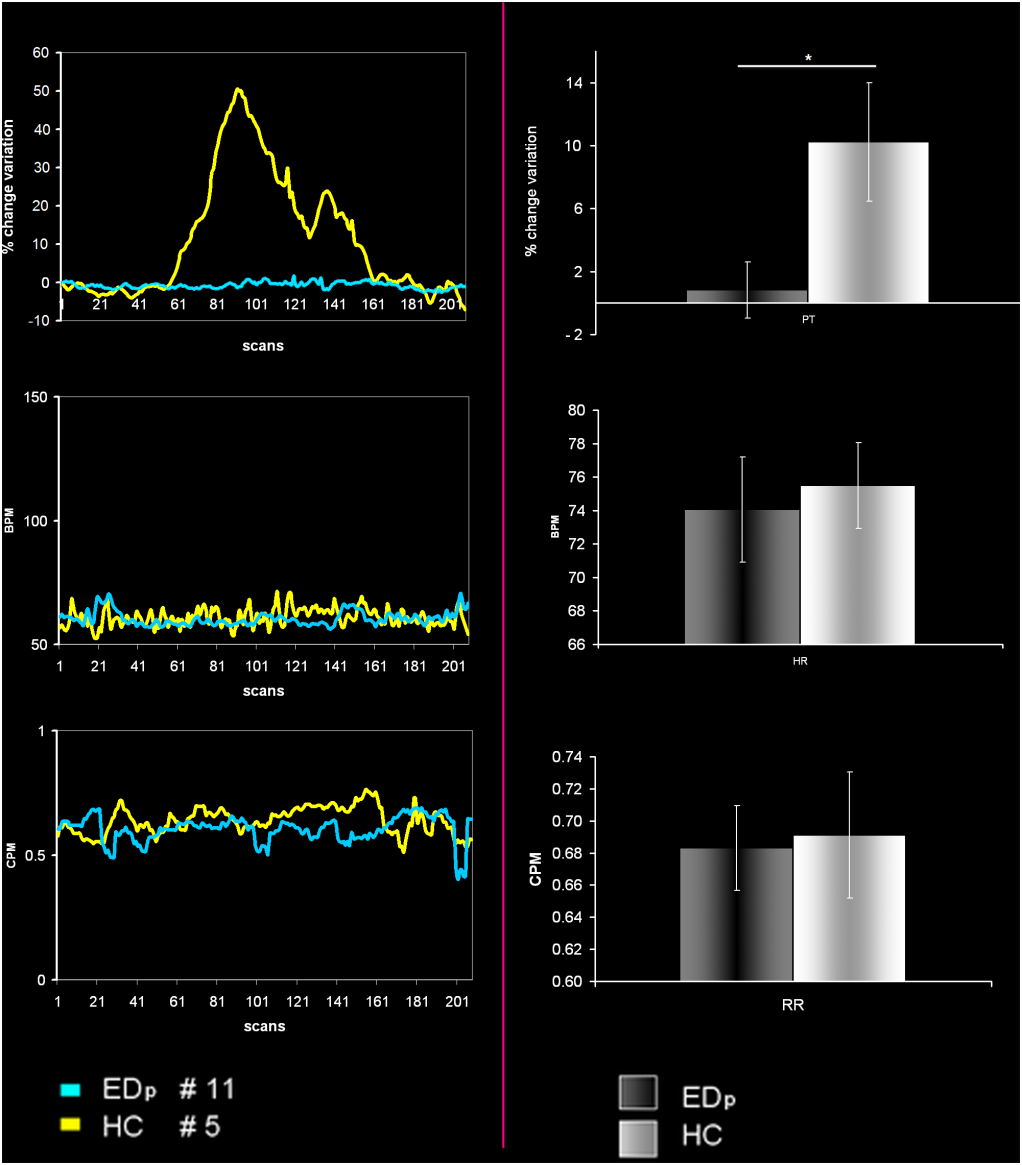
Physiological results. Left side: Example of time courses of penile reaction as recorded by the penile tumescence measuring device, heart and respiratory rate respectively for the HC number 5 and EDp number 11. Right side: histograms show averaged between group differences in penile tumescence, heart and respiratory rate respectively. Only penile tumescence shows significant differences with p<0.05. Vertical bars denote standard errors of mean (SEM).

Penile tumescence showed a significant increase only in HC group. No significant between-groups differences were observed for heart and respiratory rate (Fig. 1 and Table 1).

### Spatial pattern of networks

**Figure 2:**
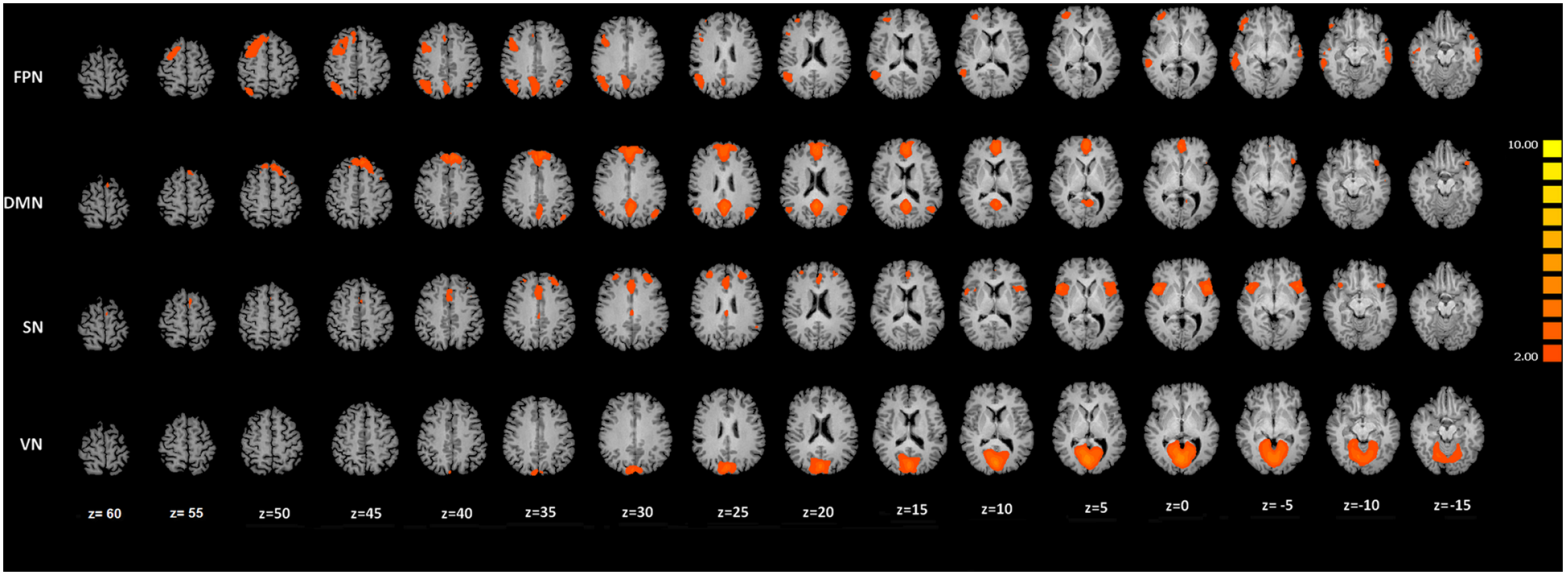
ICNs observed pooling both groups. Spatial patterns observed during erotic video clip presentation. Fronto-Parietal Network (FPN), Default-Mode Network (DMN), Salience Network (SN) and Visual Network (VN). Maps are overimposed on a Talairach atlas and are in radiological convention with a threshold of Z = 2.

ICA group classification revealed a typical spatial pattern in each network in both EDp and HC group. Our procedure for IC classification produced consistent networks [26], [46], [47]–[52], which are illustrated in Fig. 2.

The brain networks identified at group-level were: i) DMN, ii) FPN right lateralized, iii) SN, and iv) VN. We did not observe: i) AN, ii) CEN and iii) SMN.

Table 2 provides a list of the brain regions in each network, along with the Talairach coordinates of the mean peaks foci and the associated Brodmann areas (BA).

**Table 2:** Brain areas of the five networks for the two groups.

Default Mode Network						
Cluster	Hemisphere	BA	x	y	z	*t-value*
Inferior Parietal Lobe/Supramarginal gyrus	R	40	50	−59	24	1.55
Medial Prefrontal Cortex	L	10/32	−4	49	21	3.22
Posterior Cingulate cortex	L	31	−1	−53	21	3.37
Inferior Parietal Lobe	L	40	−49	−62	27	2.05
Inferior Frontal Gyrus	L	45	−43	19	−6	1.51
**Fronto Parietal Network**						
**Cluster**	**Hemisphere**	**BA**	**x**	**y**	**z**	***t-value***
Superior Parietal Lobe/Intraparietal Sulcus	R	7	38	−62	48	1.39
Middle Frontal Gyrus	R	9	44	7	42	1.12
Precuneus	R	7	2	−65	36	1.21
Anterior Frontal cortex	R	10	32	52	9	0.96
Inferior temporal Gyrus	R	20	59	−35	−3	0.96
Anterior Temporal Pole	L	38	−46	−13	−15	0.75
Middle Frontal Gyrus	L	8	−28	10	51	0.72
Superior Parietal Lobe	L	7	−43	−59	39	0.83
**Salience Network**						
**Cluster**	**Hemisphere**	**BA**	**x**	**y**	**z**	***t-value***
Anterior Insula/Inferior Frontal Gyrus	R	13	44	13	0	2.76
Anterior Insula	L	13	−43	10	0	2.61
Superior Frontal Gyrus	R	8	29	49	27	1.63
Anterior Cingulate Cortex	R	32	2	31	30	1.95
Middle/Posterior Cingulate Cortex	L	23	1	−23	27	1.57
Superior Frontal Gyrus	L	8	−7	19	57	1.40
Medial Frontal Gyrus	L	46	−31	46	30	1.94
**Visual Network**						
**Cluster**	**Hemisphere**	**BA**	**x**	**y**	**z**	***t-value***
Visual Cortex	R	17/18/19	2	−71	3	5.03

Brain regions are listed with the Talairach coordinates (x: left-right; y: anterior-posterior; z: dorsal-ventral) of the peaks of the cluster and the corresponding t values. P-values are less than 10^−6^.

Abbreviations: BA: Brodmann’s area; L: left; R: right.

**Figure 3:**
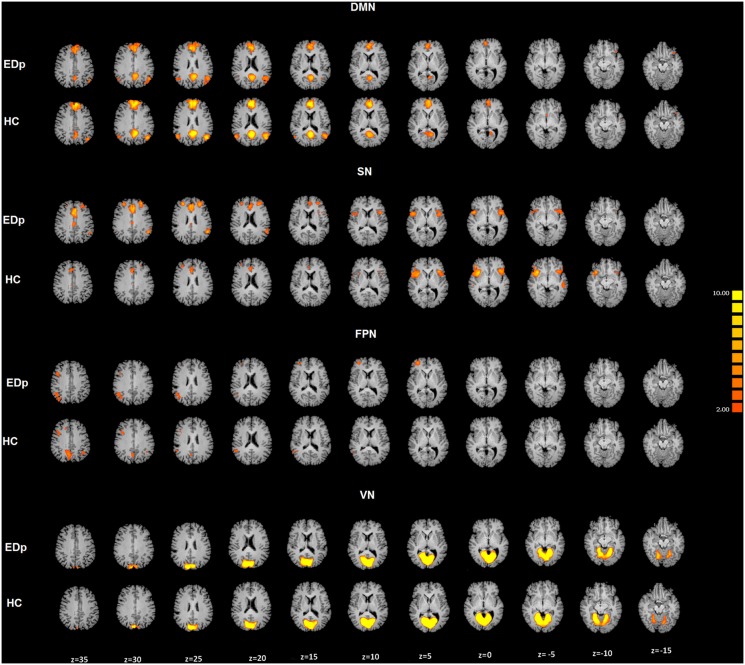
DMN, SN, FPN and VN cortical representation of group-level networks in the two groups. Top: ED patients; Down: Healthy Controls.

Among the resulting networks, the DMN and the SN showed between group differences (Fig. 3).

The DMN [26], [52] is composed by the following nodes: the Posterior Cingulate Cortex (PCC), the Precuneus (PCUN), the medial prefrontal cortex (mPFC) and two bilateral nodes observed at level of the Inferior Parietal Lobes (IPL). The SN [31] is composed by three principal nodes in correspondence of the bilateral Insulae and the ACC. The two-tailed t-test revealed significant differences in the DMN and the SN functional connectivity between the two groups. EDp group showed significant decreased Z values, indicating intrinsic connectivity levels, for the DMN with t(33) = −4.04 and p<0.01 corrected for multiple comparisons, while for the SN we observed decreased Z values with t(33) = −4.73 and p<0.01 corrected for multiple comparisons.

**Figure 4:**
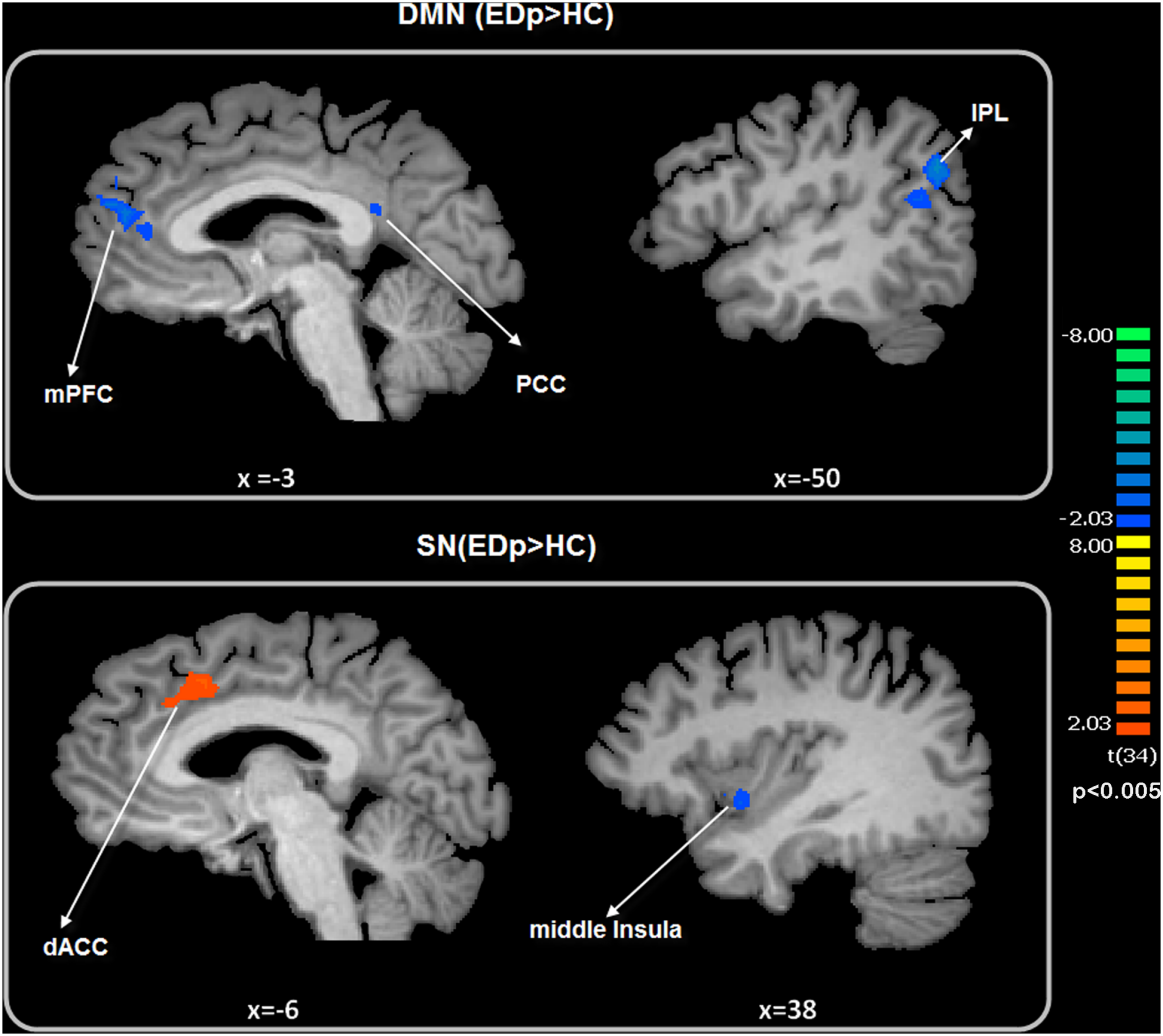
DMN and SN: between group differences. Top: DMN; Down: SN. Maps are overimposed on a Talairach atlas and are in radiological convention (p<0.05). Between groups differences are assessed by means of a voxel wise one–way ANOVA.

Moreover, a voxel wise one-way ANOVA performed on the DMN map, contrasting EDp>HC, showed a significant decreased value of connectivity in correspondence of the mPFC, the PCC/PCUN, and the left IPL. For the SN, voxel wise ANOVA showed a significant increase of intrinsic connectivity in correspondence of the dorsal ACC, while a significant decrease was observed in correspondence of the right middle Insular cortex/Claustrum (Fig. 4 and table 3).

**Table 3:** Between group results.

Default Mode Network				
	M ± ES			
Clusters	EDp	HC	t-value	*p*
Left Inferior Parietal Lobe	1±0.28	2.15±0.28	−2.83	0.007
medial Prefrontal Cortex	1.61±0.3	3.16±0.36	−3.2	0.002
Posterior cingulate Cortex/Precuneus	0.34±0.29	1.7±0.38	−2.71	0.02
**Salience Network**				
	**M ± ES**			
**Clusters**	**EDp**	**HC**	**t-value**	***p***
right middle Insula/Claustrum	−1.49±0.32	−0.30±0.16	−3.40	0.001
dorsal Anterior Cingulate Cortex	−0.36±0.14	−1.45±0.28	3.26	0.002

Data are presented in terms of mean score (M) and standard errors of means (SEM) in EDp and HC groups. Statistical comparisons between the two groups are provided.

The correlation analysis was performed between the behavioural measures of sexual function as measured by SAI-E and the Z values observed in the nodes that showed between group differences. This analysis was carried out in order to observe the specific linear relation of each node to sexual behaviour.

For the EDp group, a positive linear correlation between SAI –E (Excitation subscale) and the left IPL Z values was observed (r = 0.60, p<0.05 uncorrected).

## Discussion

In the last years, fMRI studies have shown that human sexual arousal is a complex set of sensory, cognitive and emotional processes [4]–[18]. This complexity appears to be reflected in the brain processes underlying sexual arousal, elicited by the viewing of erotic material. The present study investigated the modification of brain networks in patients with Psychogenic ED during free viewing of an erotic video clip. The ICA presents the advantage of disentangling brain activity, concurrent to the vision of complex and dynamic cinematographic material, in a set of spatially independent brain networks. In this study we used the SogICA algorithm for the group analysis instead of simpler group ICA methods. As described by Esposito et al. [24], the proposed SogICA approach is less sensitive to the presence of not homogenous sources of differences in the independent components maps structure across subjects. In general, both predictable (e.g., gender, age, etc.) as well as not easily predictable factors can contribute to bias the group ICA model estimation. This could be the case for our data that include two different groups (patients and controls), motivating the choice of a robust method like SogICA.

In the present study, the stimulus selection has been made prior to the fMRI experiment by a group of healthy men. This was performed in order to compare the different responses of the participants during the experiment. Pamilo and colleagues selected, and presented to participants, only one stimulus [21]. Moreover similar procedures were used in previously published activation studies that investigated sexual arousal [4]–[7].

During the experimental session, we observed significant between group difference in penile erectile response, with no differences in heart and respiratory rates respectively (Fig. 1). Our results are in line with previous studies [11].

The performed ICA showed that the DMN, the FPN, the VN, and the SN were spatially consistent across ED patients and healthy controls (Fig. 2). Our results are consistent with previous rsfMRI studies [31], [46], [47] and natural vision studies of brain networks [21].

Among the four observed brain networks, functional connectivity was significantly different in the DMN, that showed a decreased functional connectivity in the EDp group. While the SN, in EDp compared to HC, showed a decreased functional connectivity in the right Insula and increased connectivity in the ACC.

Our results are consistent with the previous findings on activation of specific brain regions in EDp when compared to HC. Particularly, activation studies provided evidences for specific alterations in regions involved in cognitive and emotional components of sexual arousal [4], [5]. Our results suggest that the different responses, observed in EDp, may be related to specific network dysfunction.

The spatial pattern of the obtained DMN is in agreement with those mapped in previous task and rsfMRI studies [26], [48], [49]. The DMN is an anatomically defined brain system that usually activates when individuals are not focused on the external environment [51], [52]. The observed DMN involves the PCC/pCUN, the mPFC, and the IPL. The Figure 3 depicts a reduced DMN functional connectivity observed in EDp. In particular, this group showed decreased levels of the intrinsic connectivity in the mPFC, the PCC/PCUN and the left IPL. According to Buckner [51] the DMN can be divided in two subsystems. The first is composed by the Hippocampus and the Parahippocampus regions and appeared to be involved in memory processes. The second subsystem involved the PCC, the IPLs, and the ventral mPFC. This subsystem is usually active during tasks of self relevant mental simulation. According to this view, the DMN takes part in understanding and interpreting other’s emotional status, in empathy processing and in self relevant mental simulation [51], [52]. Indeed, healthy subjects may be engaged in the simulation of the actions and emotions related to the sexual context than EDp and showed higher levels of connectivity in correspondence of the PCC/PCUN, the mPFC, and the left IPL (Fig. 4). The PCC/PCUN is the DMN hub and is usually involved in autobiographical and emotional memories [53]–[55]. The mPFC has been considered important for the regulation of emotions in general [56].

Such type of processes may be reflected in the differences observed in the mPFC that is supposed to provide information from past experiences in the form of memories during the construction of self-relevant mental simulation. Particularly, the mPFC has been related to social cognition, involving the monitoring of psychological states, and the mentalizing about the psychological states of others. Deactivation of the mPFC was found to be negatively related with erectile response in healthy subjects during visual sexual stimulation [57]. Moreover, activation in the mPFC was related to general arousal, to the self–relatedness of visual erotic stimuli, and in mechanisms mediating erectile response [15], [57], [58]. Thereby, patients showed a decreased monitoring of general arousal states with low levels of hedonic experience derived from visual erotic stimulation.

Between group differences were also found in correspondence to the left IPL. The Parietal Lobes seem to be involved in attentional and intentional processes. According to Mouras [14], activation of this region during visual sexual stimulation highlights an increased attention to sexual targets and belongs to the cognitive component of sexual arousal processing. Moreover, there is evidence that the left IPL is a component of a system implicated in visuospatial representation of bodies [59]. Remaining on this topic, there are some evidence indicating that the right IPL is crucial in the process of self/other distinction [60], [61]. However, according to Decety [62] the IPL is involved in motor imagery. Similarly, activation in the IPL during visual erotic stimulation has been related to the desire to perform similar sexual action to those depicted in the video clips [4].

Another important result was the between group differences observed in the SN. Figure 2 and table 2 showed the consistent pattern of the observed SN for both groups. The pattern of the observed SN involved the ACC and the bilateral Insular cortices in line with previous studies. The SN is involved in the integration of highly-processed sensory data with the visceral, the autonomic, and the hedonic markers allowing to the organism to make a decision [31]. In our study, we observed a different involvement of the principal nodes of the SN for the two groups. Particularly, ED patients showed a decreased level of the intrinsic connectivity in correspondence of the right middle Insula. This region has been found to be involved in different features of sexual arousal. Arnow and colleagues [6] observed that activity of the Insular cortex was related to recognition of erection, while Ferretti et al. [7] hypothesized the involvement of the Insula in mechanisms responsible for sustained penile response to erotic stimuli. Moreover, the right middle Insula is relevant for mechanisms related to both onset and sustained erection [11].

Conversely, ED patients showed higher levels of connectivity in the ACC than HC. Dorsal ACC is one of the regions involved in the bioregulation [63], the respiration [64] and the autonomic arousal states [65]. Moreover, epileptic seizures observed in the ACC, are accompanied by genital automatisms [66]. According to Abler and colleagues (2011), sexual dysfunction has been related to decreased activation in BA 24/32 [67].

## Conclusion

In summary our results showed that free viewing of erotic clip and ICA allowed the decomposition of the brain processes underlying normal and abnormal male sexual behaviour. The sexual behaviour is composed by autonomical, cognitive and emotional components that are thought to be related to a set of brain regions observed during previous activation studies. Our results highlighted abnormal brain response at network level in Psychogenic ED patients. These results showed how Psychogenic ED was related to aberrant functional connectivity in high-level network processing such as the DMN and the SN. Particularly, Psychogenic ED seems to be related to self relevant mental simulation, and in general low empathy for other’s sexual activities as well as regulation of emotions, given the decreased levels of connectivity in the DMN nodes. On the contrary, for the SN, patients showed a decreased recognition of autonomical arousal changes as suggested by the decreased connectivity in the Insula and increased connectivity in the ACC.

## References

[1] Wespes E, Amar E, Hatzichristou D, Hatzimouratidis K, Montorsi F (2005) Guidelines on Erectile Dysfunction. European Association of Urology. Available: http://www.uroweb.org/guidelines/online-guidelines/. Accessed 2014 Jul 26.

[2] Rosen RC (2001) Psychogenic erectile dysfunction: classification and management. Urologic Clinics of North America 28(2): 269–278. 11402580 10.1016/s0094-0143(05)70137-3

[3] Shamloul R, Ghanem H (2013) Erectile dysfunction. The Lancet 381(9861): 153–165. 10.1016/S0140-6736(12)60520-0 23040455

[4] Stoléru S, Grégoire MC, Gérard D, Decety J, Lafarge E, et al (1999) Neuroanatomical correlates of visually evoked sexual arousal in human males. Arc Sex Behav 28: 1–21. 10.1023/a:1018733420467 10097801

[5] Redouté J, Stoléru S, Grégoire MC, Costes N, Cinotti L, et al (2000) Brain processing of visual sexual stimuli in human males. Hum Brain Mapping 11: 162–177. 10.1002/1097-0193(200011)11:3<162::AID-HBM30>3.0.CO;2-A PMC6871964 11098795

[6] Arnow BA, Desmond JE, Banner LL, Glover GH, Solomon A, et al (2002) Brain activation and sexual arousal in healthy, heterosexual males. Brain 125: 1014–1023. 11960892 10.1093/brain/awf108

[7] Ferretti A, Caulo M, Del Gratta C, Di Matteo R, Merla A, et al (2005) Dynamics of male sexual arousal: distinct components of brain activation revealed by fMRI. Neuroimage 26: 1086–1096. 15961048 10.1016/j.neuroimage.2005.03.025

[8] Montorsi F, Perani D, Anchisi D, Salonia A, Scifo P, et al (2003) Apomorphine-induced brain modulation during sexual stimulation: a new look at central phenomena related to erectile dysfunction. Int J Impot Res. 15(3): 203–209. 10.1038/sj.ijir.3900999 12904807

[9] Borg C, de Jong PJ, Georgiadis JR (2012) Subcortical BOLD responses during visual sexual stimulation vary as a function of implicit porn associations in women. Social Cognitive and Affective Neuroscience. doi:10.1093/scan/nss117. 10.1093/scan/nss117 PMC3907922 23051899

[10] Bocher M, Chisin R, Parag Y, Freedman N, Meir Weil Y, et al (2001) Cerebral activation associated with sexual arousal in response to a pornographic clip: A 15O-H2O PET study in heterosexual men. Neuroimage 14: 105–117. 11525320 10.1006/nimg.2001.0794

[11] Cera N, Di Pierro ED, Sepede G, Gambi F, Perrucci MG, et al (2012) The role of left superior parietal lobe in male sexual behavior: dynamics of distinct components revealed by fMRI. The Journal sex. med. 9: 1602–1612. 10.1111/j.1743-6109.2012.02719.x 22510246

[12] Kim TH, Kang HK, Jeong GW (2013) Assessment of Brain Metabolites Change during Visual Sexual Stimulation in Healthy Women Using Functional MR Spectroscopy. The Journal of Sexual Medicine 10: 1001–1011. 23347356 10.1111/jsm.12057

[13] Beauregard M, Lévesque J, Bourgouin P (2001) Neural correlates of conscious self-regulation of emotion. J Neurosci 21(18): RC165. 11549754 10.1523/JNEUROSCI.21-18-j0001.2001 PMC6763007

[14] Mouras H, Stoléru S, Bittoun J, Glutron D, Pélégrini-Issac M, et al (2003) Brain processing of visual sexual stimuli in healthy men: a functional magnetic resonance imaging study. Neuroimage 20(2): 855–869. 14568457 10.1016/S1053-8119(03)00408-7

[15] Karama S, Lecours AR, Leroux JM, Bourgouin P, Beaudoin G, et al (2002) Areas of brain activation in males and females during viewing of erotic film excerpts. Hum Brain Mapping 16(1): 1–13. 10.1002/hbm.10014 PMC6871831 11870922

[16] Hamann S, Herman RA, Nolan CL, Wallen K (2004) Men and women differ in amygdala response to visual sexual stimuli. Nature neuroscience 7(4): 411–416. 15004563 10.1038/nn1208

[17] Holstege G, Georgiadis JR, Paans AM, Meiners LC, van der Graaf FH, et al (2003) Brain activation during human male ejaculation. J.Neurosci 23(27): 9185–9193. 14534252 10.1523/JNEUROSCI.23-27-09185.2003 PMC6740826

[18] Georgiadis JR, Farrell MJ, Boessen R, Denton DA, Gavrilescu M, et al (2010) Dynamic subcortical blood flow during male sexual activity with ecological validity: a perfusion fMRI study. Neuroimage 50: 208–216. 20006720 10.1016/j.neuroimage.2009.12.034

[19] Cera N, Delli Pizzi S, Di Pierro ED, Gambi F, Tartaro A, et al (2012) Macrostructural Alterations of Subcortical Grey Matter in Psychogenic Erectile Dysfunction. PLoS ONE 7(6): e39118. 22723943 10.1371/journal.pone.0039118 PMC3377616

[20] Hasson U, Nir Y, Levy I, Fuhrmann G, Malach R (2004) Intersubject synchronization of cortical activity during natural vision. Science 303(5664): 1634–1640. 15016991 10.1126/science.1089506

[21] Pamilo S, Malinen S, Hlushchuk Y, Seppä M, Tikka P, et al (2012) Functional Subdivision of Group-ICA Results of fMRI Data Collected during Cinema Viewing. PLoS ONE 7(7): e42000. 22860044 10.1371/journal.pone.0042000 PMC3408398

[22] Bordier C, Puja F, Macaluso E (2013) Sensory processing during viewing of cinematographic material: computational modeling and functional neuroimaging. Neuroimage 67: 213–226. 23202431 10.1016/j.neuroimage.2012.11.031 PMC3838951

[23] Friston KJ, Holmes AP, Poline JB, Grasby PJ, Williams SC, et al (1995) Analysis of fMRI time-series revisited. Neuroimage 2(1): 45–53. 9343589 10.1006/nimg.1995.1007

[24] Esposito F, Scarabino T, Hyvarinen A, Himberg J, Formisano E, et al (2005) Independent component analysis of fMRI group studies by self-organizing clustering. Neuroimage 25: 193–205. 15734355 10.1016/j.neuroimage.2004.10.042

[25] Fox MD, Snyder AZ, Vincent JL, Corbetta M, Van Essen DC, et al (2005) The human brain is intrinsically organized into dynamic, anticorrelated functional networks. Proc Natl Acad Sci USA 102: 9673–9678. 15976020 10.1073/pnas.0504136102 PMC1157105

[26] Raichle ME, MacLeod AM, Snyder AZ, Powers WJ, Gusnard DA, et al (2001) A default mode of brain function. Proc Natl Acad Sci U S A. 98(2): 676–82. 10.1073/pnas.98.2.676 PMC14647 11209064

[27] Damoiseaux JS, Rombouts SA, Barkhof F, Scheltens P, Stam CJ, et al (2006) Consistent resting-state networks across healthy subjects. Proc Natl Acad Sci USA 103(37): 13848–13853. 16945915 10.1073/pnas.0601417103 PMC1564249

[28] De Luca M, Beckmann CF, De Stefano N, Matthews PM, Smith SM (2006) fMRI resting state networks define distinct modes of long-distance interactions in the human brain. Neuroimage 29: 1359–1367. 16260155 10.1016/j.neuroimage.2005.08.035

[29] Corbetta M, Shulman GL (2002) Control of goal-directed and stimulus-driven attention in the brain. Nat Rev Neurosci. 3(3): 201–15. 10.1038/nrn755 11994752

[30] Greicius M (2008) Resting-state functional connectivity in neuropsychiatric disorders. Curr Opin Neurol. 21(4): 424–430. 10.1097/WCO.0b013e328306f2c5 18607202

[31] Seeley WW, Menon V, Schatzberg AF, Keller J, Glover GH, et al (2007) Dissociable intrinsic connectivity networks for salience processing and executive control. J Neurosci. 27(9): 2349–2356. 10.1523/JNEUROSCI.5587-06.2007 PMC2680293 17329432

[32] Sheehan DV, Lecrubier Y, Sheehan KH, Amorim P, Janavs J, et al (1998) The Mini-International Neuropsychiatric Interview (M.I.N.I.): the development and validation of a structured diagnostic psychiatric interview for DSM-IV and ICD-10. J Clin Psychiatry. 59: 22–33. 9881538

[33] Xu J, Mendrek A, Cohen MS, Monterosso J, Simon S, et al (2007) Effect of cigarette smoking on prefrontal cortical function in non deprived smokers performing the Stroop task. Neuropsychopharmacology 32: 1421–1428. 17164821 10.1038/sj.npp.1301272 PMC2876092

[34] Rosen RC, Riley A, Wagner G, Osterloh IH, Kirkpatrick J, et al (1997) The international Index of Erectile Function (IIEF): a multidimensional scale for assessment of erectile dysfunction. Urology 49: 822–830. 9187685 10.1016/s0090-4295(97)00238-0

[35] Hoon EF, Hoon PW, Wincze JP (1976) An inventory for the measurement of female sexual arousability. Archives of Sexual Behavior 5: 291–300. 986134

[36] Hoon EF, Chambless D (1986) Sexual Arousability Inventory (SAI) and Sexual Arousability Inventory-Expanded (SAI-E). In: Davis CM, Yaber WL, editors. Sexuality-Related Measures: A Compendium. Syracuse, NY: Graphic Publishing Co.

[37] Derogatis LR (1977) The SCL-90R Manual. I: Scoring. Administration and Procedures for the SCL-90R. Baltimore. MD: Clinical Psychometrics.

[38] Spielberger C, Gorsuch RL, Lushene RE (1970) The state–trait anxiety inventory. Palo Alto, CA: Consulting Psychologists Press.

[39] Carver S, White T (1994) Behavioural inhibition, behavioural activation, and affective responses to impending reward and punishment: the BIS/BAS scales. Journal of Personality and Social Psychology 67: 319–333.

[40] Koukounas E, Over R (1997) Male sexual arousal elicited by film and fantasy matched in content. Aust. J. Psychol 49: 1–5.

[41] Friston KJ, Williams Howard R, Frackowiak RSJ, Turner R (1996) Movement related effects in fMRI time series. Magn. Reson. Med 35: 346–355. 10.1002/mrm.1910350312 8699946

[42] Hajnal JV, Myers R, Oatridge A, Schwieso JE, Young IR, et al (1994) Artifacts due to stimulus correlated motion in functional imaging of the brain. Magn. Reson. Med 31: 283–291. 10.1002/mrm.1910310307 8057799

[43] Talairach J, Tournoux P (1988) Coplanar Stereotaxic Atlas of the Human Brain. New York: Thieme.

[44] Weissenbacher A, Kasess C, Gerstl F, Lanzenberger R, Moser E, et al (2009) Correlations and anticorrelations in resting-state functional connectivity MRI: a quantitative comparison of preprocessing strategies. Neuroimage 47(4): 1408–1416. 19442749 10.1016/j.neuroimage.2009.05.005

[45] Hyvarinen A (1999) Fast and robust fixed-point algorithms for independent component analysis. IEEE Trans Neural Netw 10: 626–634. 18252563 10.1109/72.761722

[46] Mantini D, Perrucci MG, Del Gratta C, Romani GL, Corbetta M (2007) Electrophysiological signatures of resting state networks in the human brain. Proc Natl Acad Sci USA 104(32): 13170–13175. 17670949 10.1073/pnas.0700668104 PMC1941820

[47] Mantini D, Corbetta M, Perrucci MG, Romani GL, Del Gratta C (2009) Large-scale brain networks account for sustained and transient activity during target detection. Neuroimage 44: 265–274. 18793734 10.1016/j.neuroimage.2008.08.019 PMC5745806

[48] Liao W, Chen H, Feng Y, Mantini D, Gentili C, et al (2010) Selective aberrant functional connectivity of resting state networks in social anxiety disorder. Neuroimage 52(4): 1549–1558. 20470894 10.1016/j.neuroimage.2010.05.010

[49] Forman SD, Cohen JD, Fitzgerald M, Eddy WF, Mintun MA, et al (1995) Improved assessment of significant activation in functional magnetic resonance imaging (fMRI): use of a cluster-size threshold. Magn. Reson. Med 33: 636–647. 10.1002/mrm.1910330508 7596267

[50] Beckmann CF, DeLuca M, Devlin JT, Smith SM (2005) Investigations into resting-state connectivity using independent component analysis. Philos. Trans. R. Soc. Lond. B Biol. Sci. 360: 1001–1013. 10.1098/rstb.2005.1634 PMC1854918 16087444

[51] Buckner RL, Andrews-Hanna JR, Schacter DL (2008) The brain’s default network: anatomy, function, and relevance to disease. Annals NY Ac Sci. 1124: 1–38. 10.1196/annals.1440.011 18400922

[52] Shulman GL, Corbetta M, Fiez JA, Buckner RL, Miezin FM, et al (1997) Searching for activations that generalize over tasks. Human Brain Mapping 5: 317–322. 20408235 10.1002/(SICI)1097-0193(1997)5:4<317::AID-HBM19>3.0.CO;2-A

[53] Sridharan D, Levitin DJ, Menon V (2008) A critical role for the right fronto-insular cortex in switching between central-executive and default-mode networks. Proceedings of the National Academy of Sciences 105(34): 12569–12574. 10.1073/pnas.0800005105 PMC2527952 18723676

[54] Fransson P, Marrelec G (2008) The precuneus/posterior cingulate cortex plays a pivotal role in the default mode network: Evidence from a partial correlation network analysis. Neuroimage 42(3): 1178–1184. 18598773 10.1016/j.neuroimage.2008.05.059

[55] Laird AR, Eickhoff SB, Li K, Robin DA, Glahn DC, et al (2009) Investigating the functional heterogeneity of the default mode network using coordinate-based meta-analytic modeling. The Journal of Neuroscience 29(46): 14496–14505. 19923283 10.1523/JNEUROSCI.4004-09.2009 PMC2820256

[56] Fossati P, Hevenor SJ, Lepage M, Graham SJ, Grady C, et al (2004) Distributed self in episodic memory: neural correlates of successful retrieval of self-encoded positive and negative personality traits. Neuroimage 22: 1596–1604. 15275916 10.1016/j.neuroimage.2004.03.034

[57] Moulier V, Mouras H, Pélégrini-Issac M, Glutron D, Rouxel R, et al (2006) Neuroanatomical correlates of penile erection evoked by photographic stimuli in human males. Neuroimage 33: 689–699. 16962339 10.1016/j.neuroimage.2006.06.037

[58] Walter M, Bermpohl F, Mouras H, Schiltz K, Tempelmann C, et al (2008) Distinguishing specific sexual and general emotional effects in fMRI-subcortical and cortical arousal during erotic picture viewing. Neuroimage 40: 1482–1494. 18329905 10.1016/j.neuroimage.2008.01.040

[59] Buckner RL, Carroll DC (2007) Self-projection and the brain. Trends Cogn. Sci. 11: 49–57. 10.1016/j.tics.2006.11.004 17188554

[60] Felician O, Ceccaldi M, Didic M, Thinus-Blanc C, Poncet M (2003) Pointing to body parts: a double dissociation study. Neuropsychologia. 41(10): 1307–1316. 10.1016/s0028-3932(03)00046-0 12757904

[61] Ruby P, Decety J (2003) What you believe versus what you think they believe: a neuroimaging study of conceptual perspective-taking. Europ J Neurosci. 17: 2475–2480. 10.1046/j.1460-9568.2003.02673.x 12814380

[62] Decety J (1996) Do imagined and executed actions share the same neural substrate? Brain Res. Cogn. Brain Res. 3: 87–93. 10.1016/0926-6410(95)00033-x 8713549

[63] Aziz Q, Schnitzler A, Enck P (2000) Functional neuroimaging of visceral sensation. J Clin Neurophysiol 17: 604–612. 11151978 10.1097/00004691-200011000-00006

[64] Liotti M, Brannan S, Egan G, Shade R, Madden L, et al (2001) Brain responses associated with consciousness of breathlessness (air hunger). Proc Natl Acad Sci USA 98: 2035–2040. 11172071 10.1073/pnas.98.4.2035 PMC29377

[65] Critchley HD, Corfield DR, Chandler MP, Mathias CJ, Dolan RJ (2000) Cerebral correlates of autonomic cardiovascular arousal: A functional neuroimaging investigation in humans. J Physiol. 523: 259–270. 10.1111/j.1469-7793.2000.t01-1-00259.x PMC2269796 10673560

[66] Leutmezer F, Serles W, Bacher J, Gröppel G, Pataraia E, et al (1999) Genital automatisms in complex partial seizures. Neurology 52: 1188–1191. 10214741 10.1212/wnl.52.6.1188

[67] Abler B, Seeringer A, Hartmann A, Grön G, Metzger C, et al (2011) Neural correlates of antidepressant-related sexual dysfunction: a placebo-controlled fMRI study on healthy males under subchronic paroxetine and bupropion. Neuropsychopharm. 36: 1837–1847. 10.1038/npp.2011.66 PMC3154102 21544071

